# Non-antibiotic pharmaceuticals are toxic against *Escherichia coli* with no evolution of cross-resistance to antibiotics

**DOI:** 10.1038/s44259-024-00028-5

**Published:** 2024-04-15

**Authors:** Rebecca J. Hall, Ann E. Snaith, Sarah J. Element, Robert A. Moran, Hannah Smith, Elizabeth A. Cummins, Michael J. Bottery, Kaniz F. Chowdhury, Dipti Sareen, Iqbal Ahmad, Jessica M. A. Blair, Laura J. Carter, Alan McNally

**Affiliations:** 1 Institute of Microbiology and Infection, College of Medical and Dental Sciences, University of Birmingham, Birmingham, B15 2TT UK; 2 Division of Evolution, Infection and Genomics, University of Manchester, Manchester, M13 9PT UK; 3 School of Geography, University of Leeds, Leeds, LS2 9JT UK; 4 Department of Biochemistry, Panjab University, Chandigarh, 160014 India; 5 Department of Agricultural Microbiology, Aligarh Muslim University, Uttar Pradesh, 202001 India

**Keywords:** Antimicrobials, Environmental sciences

## Abstract

Antimicrobial resistance can arise in the natural environment via prolonged exposure to the effluent released by manufacturing facilities. In addition to antibiotics, pharmaceutical plants also produce non-antibiotic pharmaceuticals, both the active ingredients and other components of the formulations. The effect of these on the surrounding microbial communities is less clear. We aimed to assess whether non-antibiotic pharmaceuticals and other compounds produced by pharmaceutical plants have inherent toxicity, and whether long-term exposure might result in significant genetic changes or select for cross-resistance to antibiotics. To this end, we screened four non-antibiotic pharmaceuticals (acetaminophen, ibuprofen, propranolol, metformin) and titanium dioxide for toxicity against *Escherichia coli* K-12 MG1655 and conducted a 30 day selection experiment to assess the effect of long-term exposure. All compounds reduced the maximum optical density reached by *E. coli* at a range of concentrations including one of environmental relevance, with transcriptome analysis identifying upregulated genes related to stress response and multidrug efflux in response ibuprofen treatment. The compounds did not select for significant genetic changes following a 30 day exposure, and no evidence of selection for cross-resistance to antibiotics was observed for population evolved in the presence of ibuprofen in spite of the differential gene expression after exposure to this compound. This work suggests that these compounds, at environmental concentrations, do not select for cross-resistance to antibiotics in *E. coli*.

## Introduction

Antimicrobial resistance (AMR) is a global public health concern^1–3^. Effluent from pharmaceutical production and wastewater treatment facilities is known to contribute to AMR^4–8^, with, for example, resistance genes identified in water bodies close to pharmaceutical plants^8–13^. Manufacturing facilities typically produce more than one chemical entity, meaning waste from these sites can contain a range of biologically active chemicals including both antibiotics and non-antibiotic compounds^14^. Whilst the evolution of bacteria in response to antibiotics is well understood^15–17^, less is known about the possible impact of long-term exposure to non-antibiotic pharmaceuticals. These include both active pharmaceutical ingredients (APIs) and other compounds found in pharmaceutical formulations. Like antibiotics, non-antibiotic pharmaceuticals are ubiquitous contaminants that have been detected in water bodies globally following inadvertent release into the environment^5,18,19^. The short- and long-term effects of these compounds on the local microbial communities remain to be established.

Existing work on the effects of APIs and other non-antibiotic compounds has uncovered some species- and compound-specific activity. Vasodilators and selective norepinephrine re-uptake inhibitors, for example, have antimicrobial activity against *Escherichia coli*, *Staphylococcus aureus*, *Pseudomonas aeruginosa*, and *Candida albicans*^20^. A screen of non-antibiotic pharmaceuticals against 40 species of gut bacteria found over 200 human-targeted drugs that had a negative effect on the growth of at least one of the species tested, with the majority active against only a few strains^21^. Titanium dioxide (TiO_2_), found in a wide range of products from cosmetics to paints^22^, has been shown to have antibacterial effects on *E. coli*^23–25^, *P. aeruginosa*^23^, *S. aureus*^23–26^ and *Enterococcus faecalis*, amongst others^27,28^. In many instances, the antimicrobial properties of TiO_2_ have been evaluated as a surface coating^24,29^ rather than a suspension, the latter representing the form in which microbial communities would be exposed to waste effluent. The anti-inflammatory drug ibuprofen has been demonstrated, using disc diffusion methods, to exhibit antimicrobial activity against species, including *S. aureus* and *Bacillus subtilis*, whilst not against *E. coli* or *P. aeruginosa*^30^, further highlighting the species-specific activity of these compounds.

There has also been recent interest in the potential of non-antibiotic pharmaceuticals to influence the susceptibility of bacteria to antibiotics. Compounds including ibuprofen, diclofenac, and propranolol have been linked to enhanced uptake of antibiotic resistance genes, possibly due to an increase in cell competency and membrane permeability, and the promotion of conjugative plasmid transfer^31,32^. The antiepileptic drug carbamazepine has also been shown to promote the transfer of plasmid-encoded resistance genes via conjugation^33^. In contrast, experiments in clinically relevant species suggest that metformin, used in the treatment of type 2 diabetes, and the non-steroidal anti-inflammatory drug benzydamine can promote uptake of tetracyclines, thereby reversing a resistance phenotype in multidrug-resistant pathogens^34,35^, and antibiotic-non-antibiotic drug combinations have been suggested as possible routes for treating infections caused by such species^36^. The short- and long-term effects of exposure to these compounds on bacterial populations, therefore warrant further study; whether they have intrinsic toxicity and, if so, the mechanism of action and the likelihood that they could act as selection pressures resulting in significant genetic changes. Importantly, discovery of the latter might indicate a possible mechanism by which exposure to non-antibiotic pharmaceuticals could select for cross-resistance to antibiotics. This is, therefore, an area of research with important clinical ramifications.

Here, we aimed to investigate the short- and long-term effects of a panel of non-antibiotic pharmaceuticals on *Escherichia coli* K-12 MG1655, with an emphasis on testing at environmentally-relevant concentrations^37–44^ to examine the potential for selecting for cross-resistance to antibiotics. The compounds selected were acetaminophen (the active ingredient in the painkiller paracetamol), ibuprofen (an anti-inflammatory), propranolol (a beta-blocker used to treat heart conditions), and metformin (a medication for type 2 diabetes). Whilst not an active ingredient in pharmaceutical formulations, TiO_2_ was also selected due to its widespread use. These compounds were all found to have a degree of toxicity against this strain of *E. coli* at a range of concentrations including those of environmental relevance, with transcriptome analysis identifying upregulated genes involved in stress response and multidrug efflux during ibuprofen treatment. Through experimental evolution in the presence of environmentally relevant concentrations of these compounds, we found evolved populations displayed decreased fitness relative to the ancestral lineage when grown in the presence of the selection compound. However, analysis of hybrid assemblies of the evolved isolates found no single nucleotide polymorphisms (SNPs) between independently evolved populations, and there was no change in minimum inhibitory concentration (MIC) for a panel of antibiotics against isolates evolved in the presence of ibuprofen compared to the ancestor. Together, this suggests that the toxicity of the non-antibiotic pharmaceuticals does not exert a selection pressure sufficiently strong enough to lead to the fixation of mutations under the conditions tested, and with no observed selection for cross-resistance to antibiotics.

## Results

### Observed toxicity from pharmaceutical compounds against *E. coli*

**Fig. 1:**
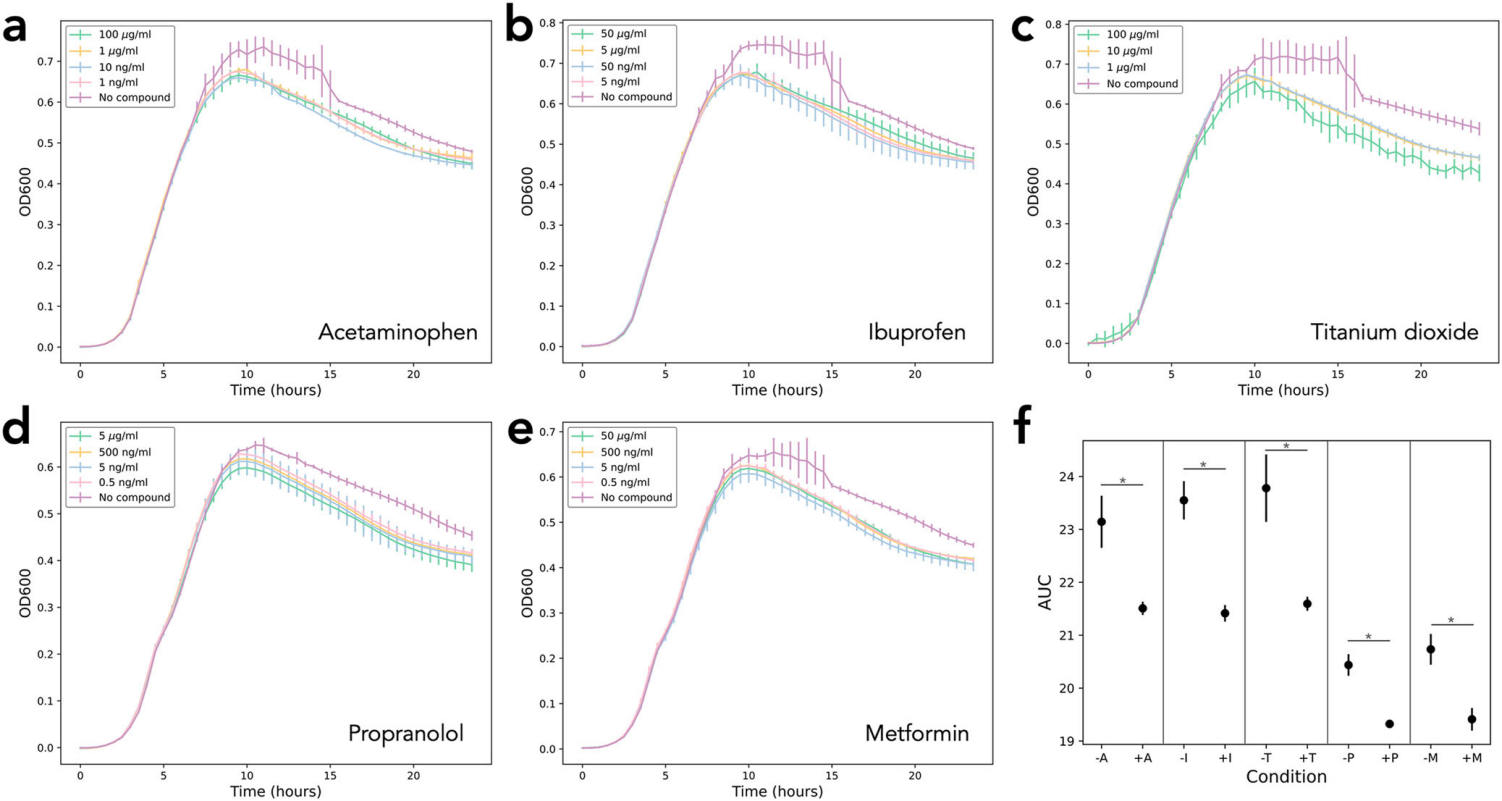
Toxicity screen of non-antibiotic pharmaceuticals against *E. coli*. Compounds **a** acetaminophen (+A), **b** ibuprofen (+I), **c** titanium dioxide (+T), **d** propranolol (+P), and **e** metformin (+M) were screened at various concentrations against *E. coli* over a 24 incubation in a 96-well plate in a microplate reader. A no-compound control (‘No compound’, purple) was included for all screens. **f** Area under the curve (AUC) values for the following concentrations are given against their representative no-compound control (-); 1 ng/mL acetaminophen, 5 ng/mL ibuprofen, 1 µg/mL titanium dioxide, 0.5 ng/mL propranolol, 0.5 ng/mL metformin. *p < 0.05, one-way ANOVA. All AUC values are shown in Supplementary Fig. 1. Measurements in triplicate, error bars depict standard deviation.

To first establish whether non-antibiotic pharmaceuticals can have observable toxicity, we screened a panel of compounds at a range of different concentrations (Supplementary Table 1) against *E. coli* K-12 MG1655 as a model organism. Acetaminophen, ibuprofen, TiO_2_, propranolol, and metformin were all found to have significant negative effects on *E. coli* growth over a 24 h incubation in comparison to the no-compound control at all concentrations tested (*p* < 0.05, one-way ANOVA, Fig. 1, Supplementary Fig. 1). The effect was predominantly noted as a reduction in the maximum OD reached. With the exception of TiO_2_ (where the highest concentration of 100 µg/mL had a larger effect than other concentrations), altering the concentration of the compounds had little effect on the resulting growth kinetics. The compounds tested can therefore negatively impact growth of *E. coli* MG1655.

### Upregulation in stress response and multidrug efflux genes in response to ibuprofen exposure

**Fig. 2:**
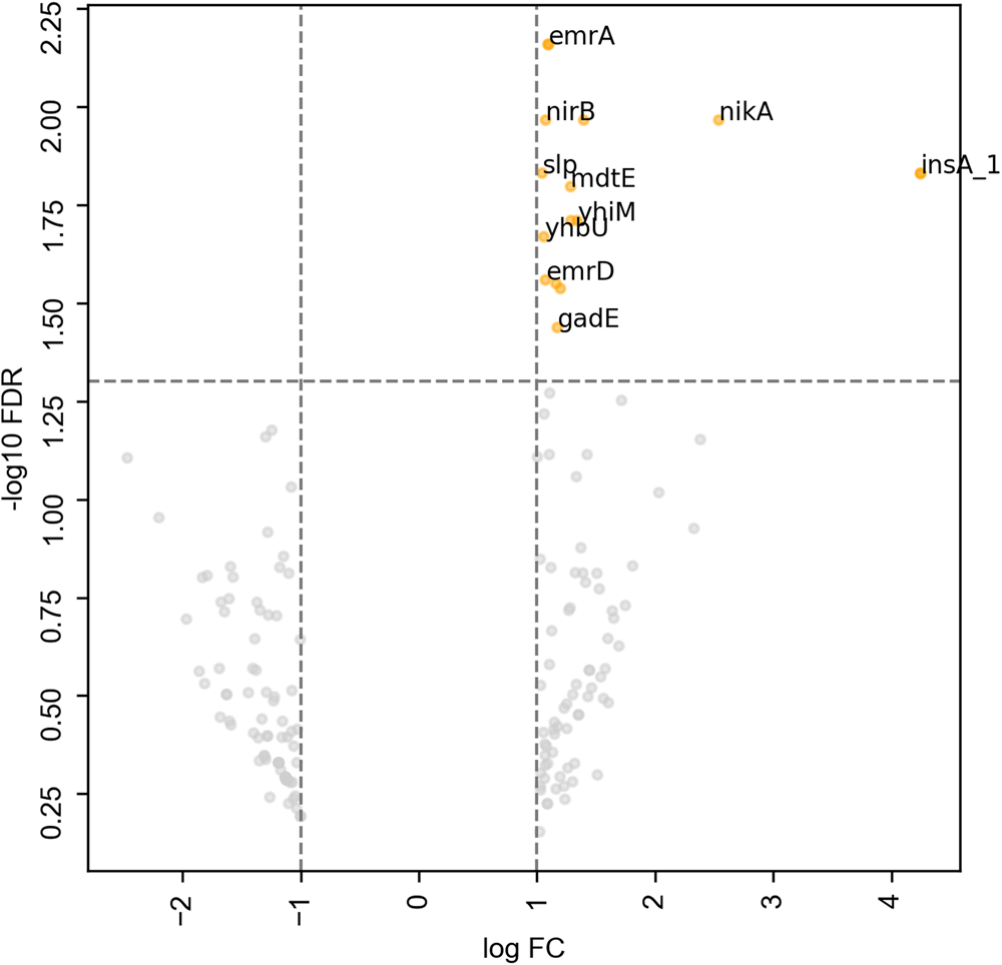
Genes differentially expressed in the presence of ibuprofen. Genes significantly upregulated (yellow) (false discovery rate [FDR] threshold of *p* < 0.05 and an absolute log fold change [FC] of at least one) in the presence of ibuprofen. Genes which had an absolute log FC of at least one but did not reach the FDR threshold are shown in grey and are considered to not be significantly differentially expressed. Selected genes are labelled.

We noted a reduction in maximum OD following exposure to the compounds. To investigate the cause of this further, we conducted transcriptomic analysis on *E. coli* populations grown in the presence and absence of 50 µg/mL ibuprofen. Ibuprofen was selected as exposure to this compound resulted in one of the larger reductions in maximum OD over a 24 h time course, and it has been linked previously to a resistance phenotype by enhancing the transfer of resistance genes^31^. We found 16 genes were significantly upregulated in the presence of ibuprofen relative to the untreated control (Fig. 2). Those with the largest log fold change that could be influencing phenotype include *insC* (4.247), *nikA* (2.539), *yhcN* (1.396), *yhiM* (1.340), *lit* (1.289) and *mdtE* (1.285) (Table 1). NikA is a periplasmic binding protein for a nickel ATP-binding cassette (ABC) transporter. The *mdtE* gene encodes the membrane fusion protein component of a multidrug efflux system. Genes involved in a second multidrug efflux transporter, *emrA* and *emrD*, are also significantly upregulated, albeit to a lesser extent. The *yhcN* gene is linked to a response to stress, and *yhiM* to acid resistance. These data suggest that the observed reduction in maximum OD following ibuprofen exposure could be attributed to the cells undergoing a stress response or actively exporting the compound.

**Table 1:** *E. coli* genes upregulated significantly in the presence of ibuprofen, their function as assigned by Prokka, and the average (of biological triplicate) log fold change (FC) when normalised against *E. coli* grown in the absence of ibuprofen

Gene	Function	log FC
insA	Is*2* element protein	4.247
insA	Is*2* element protein	4.247
nikA	Nickel ABC transporter - periplasmic binding protein	2.539
yhcN	Stress-induced protein	1.396
yhiM	Inner membrane protein with a role in acid resistance	1.34
lit	Cell death peptidase; phage exclusion; e14 prophage	1.289
mdtE	MdtEF-TolC multidrug efflux transport system - membrane fusion protein	1.285
yhiD	Putative Mg(2+) transport ATPase	1.199
gadE	DNA-binding transcriptional activator	1.172
bhsA	Outer membrane protein involved in copper permeability, stress resistance and biofilm formation	1.161
aegA	Putative oxidoreductase, Fe-S subunit	1.099
emrA	EmrAB-TolC multidrug efflux transport system - membrane fusion protein	1.093
nirB	Nitrate reductase, large subunit	1.073
emrD	Multidrug efflux transporter	1.072
yhbU	Putative peptidase (collagenase-like)	1.059
slp	Starvation lipoprotein	1.044

### Co-exposure to ibuprofen and antibiotics does not alter MICs for *E. coli* MG1655

Microbial communities residing in or near industrial wastewater will be exposed to a cocktail of antibiotic and non-antibiotic compounds. The presence of a nonantibiotic pharmaceutical may induce a response that could alter the MIC of an antibiotic during co-exposure. To assess this, *E. coli* MG1655 and ATCC 25922 (as a control strain) were co-exposed to ibuprofen plus one of the following antimicrobial agents; ethidium bromide, ampicillin, ciprofloxacin, chloramphenicol, trimethoprim, and colistin. No change in MIC was observed for *E. coli* MG1655 for any ibuprofen-antimicrobial pair, with FIC scores indicating no synergy or antagonism (Table 2). This suggests that in laboratory strains of *E. coli*, co-exposure to ibuprofen alongside an antibiotic does not alter the resistance profile.

**Table 2:** MICs (mg/L) for six antimicrobial agents against the MG1655 ancestral lineage, the MG1655 strain evolved in the presence of ibuprofen, and an ATCC 25922 control strain (n=3, one biological replicate each, modal MIC value given with the exception of ciprofloxacin against MG1655 evolved where the median value is given)

Agent	MIC ancestor	MIC evolved	MIC ATCC 25922	CB MIC ancestor	FIC ancestor	CB MIC ATCC 25922	FIC ATCC 25922
Ethidium bromide	512	512	256	512	2	256	2
Ampicillin	4	8	8	8	3	8	2
Ciprofloxacin	0.0156	0.0156	0.0156	0.0156	2	0.0156	2
Chloramphenicol	8	8	4	8	2	4	2
Trimethoprim	0.25	0.25	0.25	0.5	3	0.5	3
Colistin	4	4	4	2	1.5	4	2
Ibuprofen	>200	-	>200	>200	-	-	-

Checkerboard (CB) MICs (mg/L) for *E. coli* MG1655 and ATCC 25922 co-exposed to ibuprofen plus an antimicrobial agent, one of; ethidium bromide, ampicillin, ciprofloxacin, chloramphenicol, trimethoprim, or colistin (*n* = 3, one biological replicate each, modal MIC given). Fractional Inhibitory Concentration (FIC) scores calculated whereby 0.5–4 indicates no synergy or antagonism. - indicates not applicable/not tested.

### Long-term exposure to non-antibiotic pharmaceuticals impacts *E. coli* growth but does not select for cross-resistance to antibiotics

**Fig. 3:**
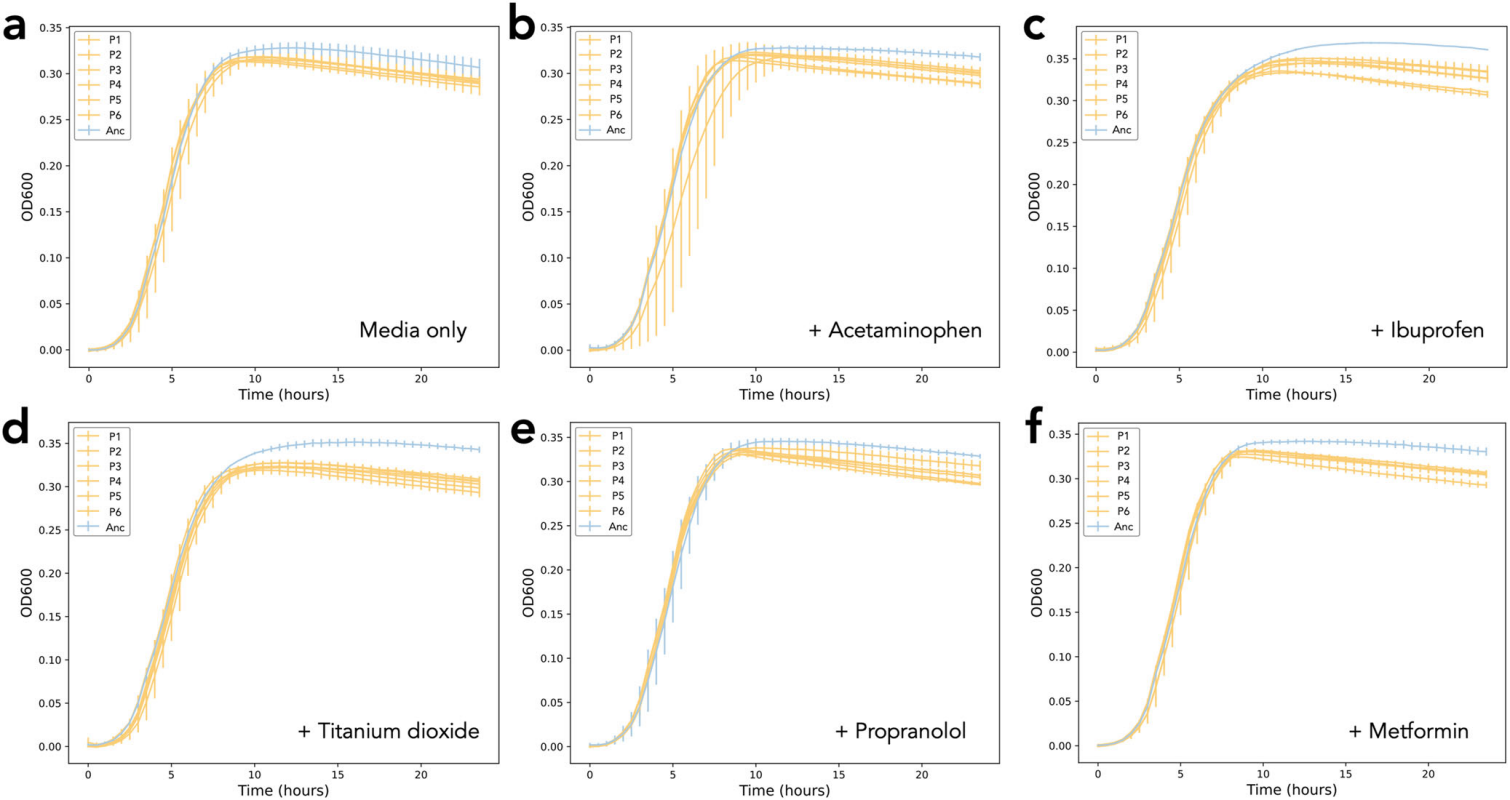
Growth of ancestor and evolved populations. Growth of evolved populations (yellow, six independent biological replicates P1-P6) in the presence of the compound in which their selection experiment was conducted in comparison to growth of the ancestral lineage (blue, Anc); **a** media-only control, **b** acetaminophen, **c** ibuprofen, **d** titanium dioxide, **e** propranolol, and **f** metformin. Measurements in triplicate, error bars depict standard deviation.

After establishing the negative impact on *E. coli* growth in the presence of selected non-antibiotic pharmaceuticals, we investigated whether this would be sufficient to act as a selective pressure during long-term exposure. We, therefore, propagated populations in the presence and absence of acetaminophen, ibuprofen, TiO_2_, metformin, and propranolol individually, passaging cells every 24 h for 30 days. The evolved populations were then screened in the presence and absence of their selection compound to assess growth in comparison to the ancestral lineage. We observed a decrease in the maximum OD readings reached by the evolved populations in their selection media in comparison to the ancestral lineage, suggesting that prolonged exposure to the compounds did not select for improved growth (Fig. 3). This difference was statistically significant in the majority of populations (*p* < 0.05, one-way ANOVA, Supplementary Fig. 2), and was most notable for the populations exposed to ibuprofen and TiO_2_ (Fig. 3). The reduction in OD observed in the no compound control was calculated to be not significant (Supplementary Fig. 2). The difference remained when the populations were exposed to 100× concentration of the compounds (Supplementary Fig. 3), and when all populations were passaged through a 7-day ‘recovery’ experiment in NB with no added pharmaceutical (Supplementary Fig. 4). This suggests therefore that the growth patterns observed in the evolved populations was not a transient, reversible effect as a result of long-term toxicity.

To establish whether there were any significant SNPs arising as a result of the selection experiments, short-read assemblies were generated for three replicates from each of the control and test conditions and the sequences analysed using Snippy. No mutations parallel between independent evolving populations were found within treatments (Supplementary Table 3). SNPs in *recQ* (ATP-dependent DNA helicase) and *ygeA* (a putative racemase) were observed in single replicates of evolved populations exposed to acetaminophen. The singular occurrence of each suggests they arose as a result of drift rather than selection, or that selection was not strong enough for them to reach fixation within the other populations. An analysis of the gene presence/absence patterns across the hybrid assembled evolved isolate genomes highlighted sequence variation in *ldrA* gene in three sequences; one control, and one each evolved in acetaminophen and TiO_2_. All had three SNPs compared to the ancestral MG1655 (Supplementary Table 2). The TiO_2_ and the control had identical SNPs, whereas the three SNPs in the acetaminophen-exposed isolate were different. Again, these variations occurred in a single replicate per condition only.

The IS*2* element *insA* was shown to be upregulated in the presence of ibuprofen. IS element transposition could be a cause of the differences in growth patterns between the ancestral and evolved isolates. To assess this, hybrid assemblies were generated and the distribution of IS elements then established using ISEScan and ISFinder. All IS elements were present in equal numbers between the ancestor and all evolved lineages. IS*2*, IS*30*, and IS*1* elements were interrogated in depth and showed no evidence of movement between any evolved isolate and the ancestor. Overall, these data suggest that although the presence of TiO_2_ and the non-antibiotic pharmaceuticals has a negative effect on the growth of *E. coli*, they do not exert selection pressure during prolonged exposure at the timescale and concentrations tested.

Given the observed upregulation in known efflux systems following ibuprofen treatment, we examined whether prolonged exposure to ibuprofen may select for cross-resistance to antibiotics. The MICs of several antimicrobials were analysed for a strain evolved in the presence of ibuprofen compared to the ancestral isolate. Ethidium bromide, ampicillin, ciprofloxacin, chloramphenicol, trimethoprim, and colistin were chosen based on the previously mentioned transcriptomic data as compounds which might logically have altered MICs as a result of ibuprofen exposure. The MICs of the evolved isolate for all antibiotics tested were either the same or within one doubling dilution of the ancestor (Table 2). This indicates that long-term exposure to ibuprofen, regardless of the differential gene expression, does not co-select for resistance to the antibiotics tested.

## Discussion

The evolution of bacteria, including *E. coli*, in response to exposure to antibiotics is well understood. The effects of short- and long-term exposure to non-antibiotic pharmaceuticals and other compounds in pharmaceutical formulations are however less clear. This includes their potential toxicity and the likelihood that their presence might select for cross-resistance to antibiotics. With increasing evidence for the presence of non-antibiotic pharmaceuticals in proximity to production plants^5,18^, it is becoming apparent that these are compounds that require further investigation into their potential to impact local microbial populations. To begin to address this, we screened a panel of four non-antibiotic pharmaceuticals and TiO_2_ against a laboratory strain of *E. coli* to assess their potential toxicity and found that all five, to various degrees, reduced the maximum OD reached by the population over a 24 h incubation. This, therefore, suggests that these compounds may, even at low concentrations (1 ng/mL acetaminophen, 5 ng/mL ibuprofen, 0.5 ng/mL metformin and propranolol, 1 µg/mL TiO_2_), have a negative effect on members of the microbial communities surrounding production plants. Our results contribute to published work on ibuprofen toxicity, whereby it was demonstrated through disc diffusion assays to not have antimicrobial activity against *E. coli*^30^.

We then uncovered 16 genes that are upregulated significantly when *E. coli* was grown in the presence of ibuprofen. Notable amongst these genes were two periplasmic adaptor proteins from different multidrug efflux systems; *mdtE* and *emrA*. MdtEF-TolC is a resistance nodulation division (RND) family pump with beta-lactams, benzalkonium chloride, macrolides, and oxazolidinones as known substrates, and EmrAB-TolC is a major facilitator superfamily (MFS) efflux pump that confers resistance to compounds including fluoroquinolones in *E. coli*^45^. Efflux pump expression is often upregulated in the presence of toxic compounds to prevent their accumulation inside the cell^46–49^. There is some existing evidence linking efflux pumps to a response to non-antibiotic pharmaceuticals. Exposing *S. aureus* to diclofenac has been shown to downregulate a putative *emrAB*-family pump^50^, which contrasts with the upregulation we observed in *E. coli* following ibuprofen exposure. The response could, therefore, be specific to the species, compound, or pump, and more work is needed to unravel this potential interaction.

Ibuprofen is an example of a partial proton motive force (PMF) uncoupler that can inhibit the function of RND and MFS pumps^51^. The nitrite reductase *nirB* was identified as another significantly upregulated gene. Nitrate reduction has been shown to enhance bacterial survival in the presence of agents that dissipate PMF^50^. This, therefore, provides tentative support to a hypothesis that *E. coli* may be using nitrate reduction to ameliorate the dissipation of PMF in the presence of ibuprofen, enabling the function of the RND and MFS pumps.

Ibuprofen exposure also resulted in the upregulation of several genes linked to a response to stress, including *yhcN*, the inner membrane protein *yhiM*, and the outer membrane protein *bhsA*. Existing research has shown a *bhsA* mutant of *E. coli* to be more sensitive to a variety of stressors including acid^52^, and *yhcN* has been linked to cytoplasm pH stress^53^. Additionally, the gene observed to be upregulated to the greatest extent in our data was *insA*, and IS*2* and other IS elements are known to be upregulated in response to stress^54,55^. An upregulation of stress response genes in response to non-antibiotic pharmaceuticals has been noted previously in *A. baylyi*, where the uptake of antibiotic resistance genes was shown to be facilitated by the presence of compounds including ibuprofen and propranolol^31^. There, analysis including transcriptomics linked the observation to increased stress and the over-production of reactive oxygen species, amongst other characteristics.

Our data suggest that the non-antibiotic components within pharmaceutical production waste may affect the local microbial communities, as over time the toxicity observed here may deplete species or genera within the communities, altering their composition. Despite the observed reduction in maximum OD, we found that prolonged exposure to this panel of non-antibiotic pharmaceuticals did not result in significant genetic changes across multiple independent populations. It is possible that the stress induced by the compounds was dealt with sufficiently by, for example, the upregulation of efflux pumps, thereby reducing the selection pressure, or that the reduced OD was not due to changes in carrying capacity but rather due to morphological changes following induction of stress responses. We also found no evidence of synergy when the ancestral strain was co-exposed to ibuprofen and one of a panel of antibiotics. Additionally, when the same panel of antibiotics was tested against the ancestor and the strain evolved in the presence of ibuprofen, there was no evidence of altered MICs in the latter that would indicate selection for cross-resistance to antibiotics. This is a reassuring initial investigation given the large quantities of pharmaceutical production waste entering local ecosystems. Whilst the panel of compounds tested here is small, they are commonly used and found consistently in water bodies, and therefore can be considered a representative sample^56–59^. Acetaminophen, ibuprofen, metformin, and propranolol have also been identified as priority pharmaceuticals in India^60^. The use of a standard laboratory strain of *E. coli* is a useful starting point, but previous work suggesting that the activity of non-antibiotic pharmaceuticals may be strain-specific^21^ underscores the need for broad-spectrum testing before definitive conclusions can be drawn. Variations in concentrations over time as a result of effluent changes, dry seasons, and climate change should also be considered, and there is a need to extend this research to encompass production waste as a holistic entity against environmentally relevant populations.

## Methods

### Strains and growth conditions

To measure potential toxicity of compounds, *E. coli* K-12 MG1655 was streaked from a glycerol stock on to a Luria Bertani (LB) agar plate (E & O Laboratories Ltd), incubated overnight at 37 °C. A single colony was then used to inoculate 5 mL LB (E & O Laboratories Ltd) in a 30 mL universal before overnight incubation at 37 °C with agitation. The overnight cultures were diluted to an optical density at 600 nm (OD600) of ~0.5 in LB. Serial dilutions of acetaminophen, ibuprofen, propranolol, and metformin were prepared as per Supplementary Table 1. These compounds were selected as both published literature and preliminary investigations identified their presence in wastewater and receiving water environments, and they are commonly used non-antibiotic pharmaceuticals^5^. Titanium dioxide (TiO_2_), in the form of nanoparticles, was also used as it is found in a wide range of products and has suggested applications in water treatment^27,61,62^. Environmentally relevant concentrations were identified following a literature search and are provided in Supplementary Table 1. A solution of 99 µL of LB + compound was added to each test well of a 96-well plate, including an LB-only control, with 1 µL of the dilute cell suspension then added. Plates were incubated for 24 h in a microplate reader (Tecan) at 37 °C with continuous double orbital shaking, with absorbance measurements (OD600) taken every 30 min in triplicate. To assess the growth kinetics of evolved populations, ten colonies were selected for incubation as representative of the population and the kinetics monitored in a microplate reader as described previously, in the presence of the compound to which they were exposed during the selection experiment. Compounds were tested at 1× and 100× selection concentrations (Supplementary Table 1) to measure whether the evolved isolates would show improved growth compared to the ancestral lineage when stressed with a higher concentration of the compound to which they had been exposed during the selection experiment.

### Genome sequencing and bioinformatics

Illumina short-read sequencing of the ancestral and evolved isolates was performed by MicrobesNG (UK). Long-read sequencing of the same isolates was performed using MinION sequencing (Oxford Nanopore Technologies, UK). Briefly, genomic DNA was extracted from overnight cultures using the Monarch Genomic DNA Purification Kit (New England Biolabs). DNA was quantified using a Qubit 4 fluorometer (Invitrogen) and accompanying broad-range double-stranded DNA assay kit (Invitrogen). Sequencing libraries were prepared using SQK-LSK109 ligation sequencing kit and EXP-NBD114 native barcode expansion (Oxford Nanopore Technologies, UK), as per manufacturer instructions. Long-read sequencing was performed on a MinION sequencer using an R9.4.1 flow cell (Oxford Nanopore Technologies, UK). Base calling was conducted using Guppy (v6.0.1). Reads were filtered using Filtlong (v0.2.1) using a cut-off of 600,000,000 target bases and demultiplexed using qcat (v1.1.0). Hybrid assemblies were then generated using Unicycler (v0.4.8-beta) in bold mode. Panaroo (v1.2.10) was used to generate gene presence/absence and core gene alignment files, with the latter used to construct a maximum likelihood tree with IQ-TREE (v2.2.0.3). The tree and gene presence/absence data were visualised in Phandango^63^ to look for differential gene presence patterns across the evolved isolates. A custom ABRicate (v0.8) database was used to investigate the presence and identity of the *ldrA* gene across the evolved isolates. The presence of SNPs was analysed using snippy (v4.3.6). Potential movement of insertion sequence (IS) elements was investigated using ISEScan (v1.7.2.3), ISFinder^64^, and a custom ABRicate (v0.8) database, with sequences interrogated in Unipro UGENE (v47.0).

### Transcriptome sequencing

RNA sequencing was performed on *E. coli* grown in the presence and absence of 50 µg/mL ibuprofen in triplicate. For the control (absence) replicates, an equivalent volume of the ibuprofen solvent (ethanol) was added. For sample preparation, a single colony for each replicate was picked following overnight growth on LB agar and added to 5 mL of LB broth (Sigma-Aldrich, UK). A 100 µL suspension of each overnight culture was then transferred into 10 mL fresh LB in the presence or absence of 50 µg/mL ibuprofen, with cultures then incubated at 37 °C with agitation until an optical density at 600 nm (OD600) of ~0.9. A 1 mL sample was centrifuged for 5 min at 10,000 rpm (Eppendorf MiniSpin F-45-12-11), resuspended in 1 mL phosphate-buffered saline (VWR), and this wash step was repeated. The supernatant was aspirated and the pellet frozen prior to processing and RNA sequencing by GENEWIZ from Azenta Life Sciences (Frankfurt, Germany) using their standard RNA sequencing service. Differential gene expression was quantified using Kallisto (v0.48.0). A long-read assembly of the ancestral *E. coli*, annotated using Prokka (v1.14.6), was used as a reference. The annotated assembly was processed using genbank_to_kallisto.py (https://github.com/AnnaSyme/genbank_to_kallisto.py). GNU parallel^65^ was used for job parallelisation. Differential gene expression was analysed in Degust (v4.1.1) with a false discovery rate threshold of *p* < 0.05 and an absolute log fold change of at least 1.

### Selection experiment

The ancestral *E. coli* isolate was streaked from a glycerol stock on to an LB plate and incubated overnight at 37 °C. A single colony was used to inoculate 5 mL nutrient broth (NB) (Sigma) in a 30 mL universal, with six independent biological replicates per condition. Acetaminophen (5 ng/mL), ibuprofen (2 µg/mL), TiO_2_ (1 µg/mL), propranolol (0.5 ng/mL), and metformin (0.5 ng/mL) were tested individually, including a NB-only control. Microcosms were incubated for 24 h at 37 °C with agitation, before a 1% transfer of cell suspension into fresh media. This 1% transfer was repeated every 24 h for 30 days. After 30 days, the whole population was centrifuged at 3600 rpm (Thermo Scientific Megafuge 40 R TX-1000) for 5 min, resuspended in 1 mL 50% glycerol, and stored at −80 °C. To assess whether the populations were experiencing short-term, reversible toxicity as a result of compound exposure, ten colonies from each end-point population were selected from a UTI ChromoSelect agar plate (Millipore) and used to inoculate 5 mL NB only. The microcosms were incubated for 24 h at 37 °C with agitation, before a 1% transfer of cell suspension into fresh media every 24 h for 7 days. After 7 days, the whole population was centrifuged at 3600 rpm (Thermo Scientific Megafuge 40 R TX-1000) for 5 min, resuspended in 1 mL 50% glycerol, and stored at −80 °C.

### Minimum inhibitory concentration assay

Stocks of ethidium bromide, ampicillin, ciprofloxacin, chloramphenicol, trimethoprim, and colistin were prepared at 1000 µg/mL. Cultures of the *E. coli* MG1655 ancestor and an *E. coli* ATCC 25922 control strain were prepared by inoculating 5 mL of LB with a single colony of bacteria and incubating with agitation at 37 °C for 18 h. Iso-Sensitest broth (ISB) (Thermo Fisher Scientific) was then used throughout the assay. The overnight culture was then diluted 1:100 and working stocks of antibiotics were prepared, both in ISB. U-bottom 96-well plates were set up so that the cultures were incubated with no antibiotic and with 11 different concentrations of antibiotic ranging from 0.008 to 8 µg/mL. The first column of the 96-well plate contains the highest concentration of antibiotic and the 11th column contains the lowest concentration, with the 12th column containing no antibiotic, and 50 µL of the diluted cell suspension was added to all wells. Plates were incubated at 37 °C for 18 h and examined for growth the next day. Results were only accepted if the observed MIC for the ATCC 25922 strain was within one doubling dilution of the expected result.

### Checkerboard minimum inhibitory concentration assay

Cultures of the *E. coli* MG1655 ancestral lineage and one of the six end-point isolates evolved in the presence of ibuprofen were prepared using a single colony inoculated into LB broth before incubation overnight at 37 °C with agitation. Working stocks of ethidium bromide, ampicillin, ciprofloxacin, chloramphenicol, trimethoprim, colistin, and ibuprofen were prepared at four times the highest final concentration required by diluting in ISB. Overnight cultures were diluted 1:2000 in ISB. A 50 µL aliquot of ISB was added to all columns of a 96-well plate, and 50 µL working ibuprofen stock added to all wells of columns one and two. Starting with column two, the ibuprofen was serially diluted 1:2 across the plate up to and including column 11. A 50 µL aliquot of one antibiotic working stock was then added to all wells of row A, the 1:2 dilution repeated down the plate up to and including row G, and 50 µL removed from column 11 and row G before adding cells to keep the volume consistent. A 50 µL sample of diluted overnight culture was then added to each well and mixed gently before the plate was covered and incubated for 18 h at 37 °C static. Plates were read following incubation and the presence or absence of growth was noted.

### Statistical analyses

The area under the curve measurements was calculated using numpy.trapz in Python (v3.9.10). Significance testing was conducted using a one-way analysis of variance (ANOVA).

### Reporting summary

Further information on research design is available in the Nature Research Reporting Summary linked to this article.

## Supplementary information

Supplementary Material

Reporting Summary

## Data Availability

The datasets generated and analysed during the current study are available from NCBI BioProject with accession PRJNA1005239.
