# Supplementary material for: Non-antibiotic pharmaceuticals are toxic against Escherichia coli with no evolution of cross-resistance to antibiotics
Source: NPJ Antimicrob Resist. 2024 Apr 15;2:11. doi: 10.1038/s44259-024-00028-5 (PMC11721113; doi:10.1038/s44259-024-00028-5)
Supplement: Supplementary file 1 — Supplementary Material [file 44259_2024_28_MOESM1_ESM.pdf]

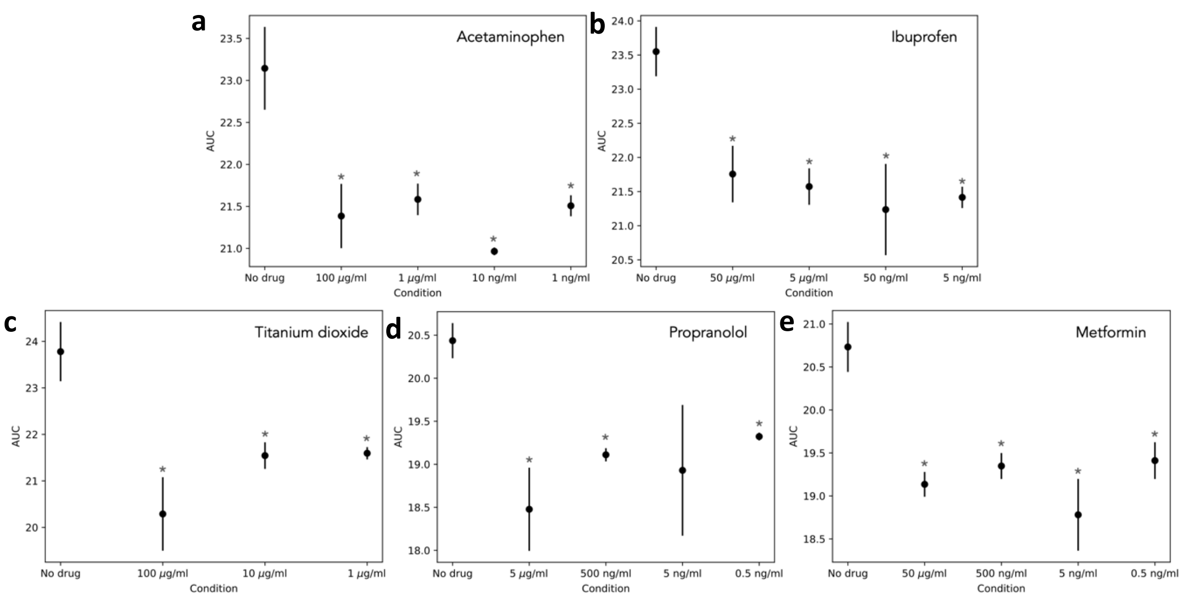

Supplementary Figure 1. Area under the cure values for toxicity screen against *E. coli*. Area under the curve (AUC) values for a toxicity screen of **a** acetaminophen, **b** ibuprofen, **c** titanium dioxide, **d** propranolol, and **e** metformin at various concentrations against *E. coli*, relating to Fig. 1. A no-compound control ('No drug') was included for each screen. * $p < 0.05$, one-way ANOVA. Measurements in triplicate, error bars depict standard deviation.

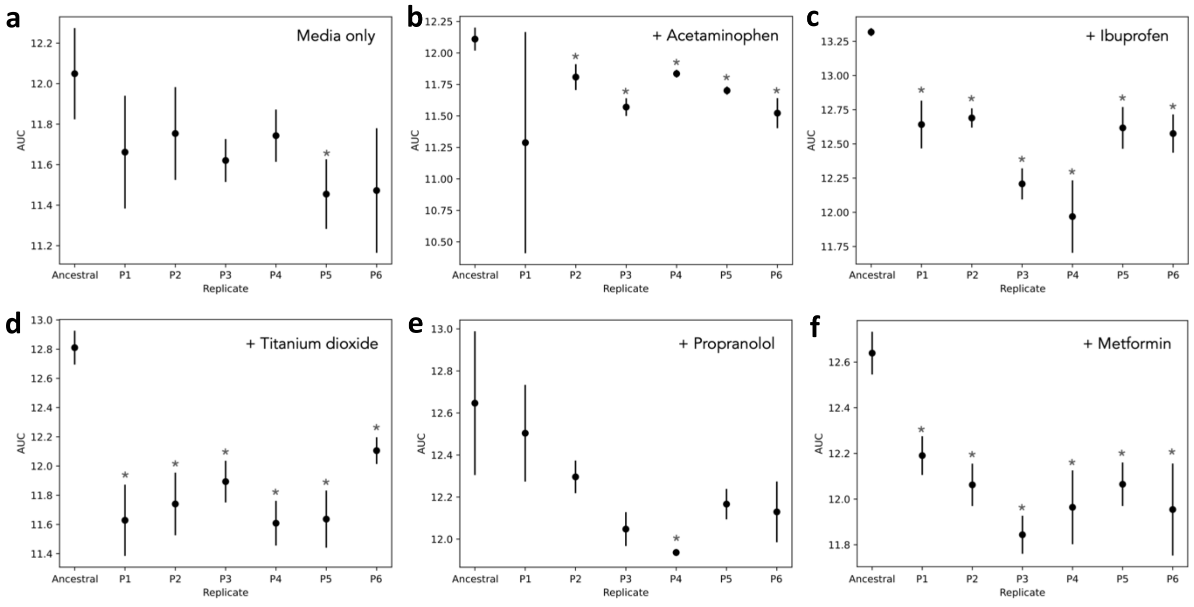

Supplementary Figure 2. Area under the curve values for ancestor and evolved populations in the presence of non-antibiotic pharmaceutical. Area under the curve (AUC) values for evolved populations (six independent biological replicates P1-P6) in a fresh sample of their respective evolution media in comparison to the ancestral lineage, relating to Fig. 3. * $p < 0.05$, one-way ANOVA. Measurements in triplicate, error bars depict standard deviation.

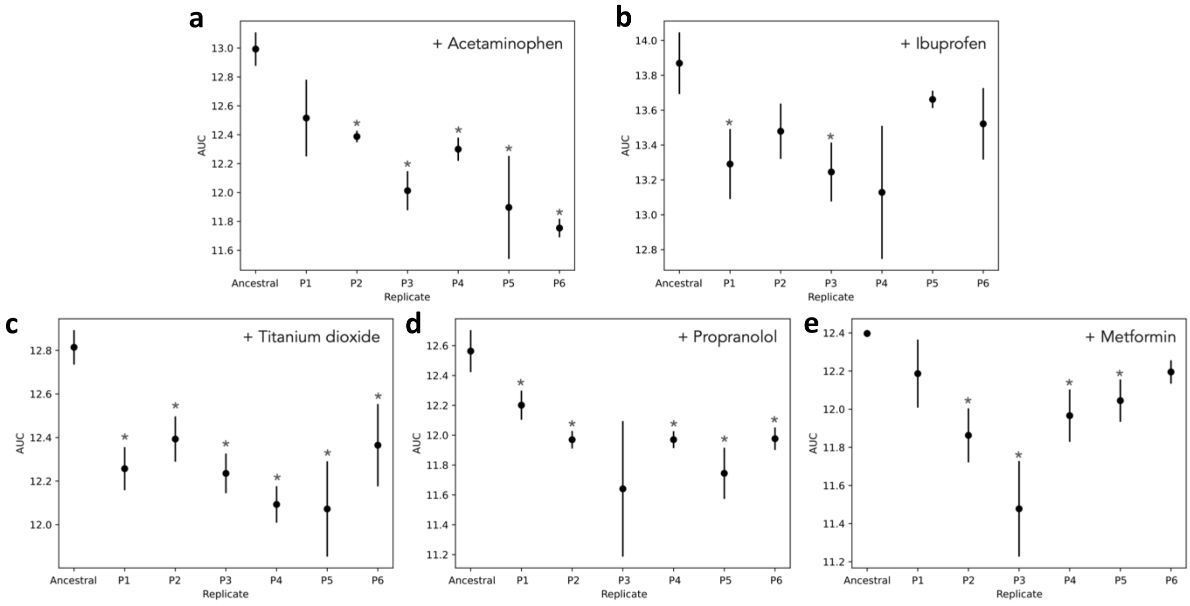

Supplementary Figure 3. Area under the curve values for ancestor and evolved populations in 100x concentration of compounds. Area under the curve (AUC) values for evolved populations (six independent biological replicates P1-P6) in the presence of 100x concentration of the compound in which their selection experiment was conducted in comparison to growth of the ancestral lineage. * $p < 0.05$, one-way ANOVA. Measurements in triplicate, error bars depict standard deviation.

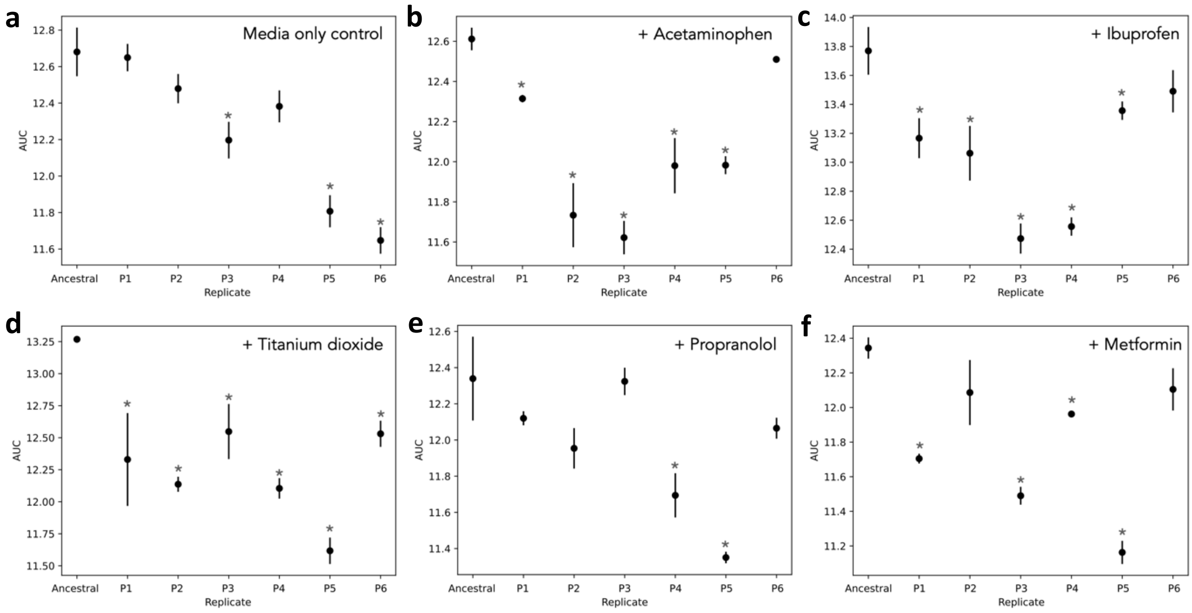

Supplementary Figure 4. Area under the curve values for ancestor and evolve populations in the presence of non-antibiotic pharmaceuticals after seven day recovery. Area under the curve (AUC) values for evolved populations (six independent biological replicates P1-P6) following serial passaging for seven days in the absence of pharmaceuticals in comparison to the ancestral lineage. * $p < 0.05$, one-way ANOVA. Measurements in triplicate, error bars depict standard deviation.

Supplementary Table 1. Non-antibiotic pharmaceuticals concentrations. Concentrations used to screen the non-antibiotic pharmaceutical compounds for toxicity against E. coli, and the final concentration chosen for selection experiments. No-compound controls were also used as comparisons for all screens. Relevant references are also included.

Compound	Conc. (screen)	Conc. (selection)	Manufacturer	Ref
Acetaminophen	100 µg/mL, 1 ug/mL, 10 ng/mL, 1 ng/mL	5 ng/mL	Merck Life Science UK Ltd	37
Ibuprofen	50 µg/mL, 5 µg/mL, 50 ng/mL, 5 ng/mL	2 µg/mL	Merck Life Science UK Ltd	38, 39
Titanium dioxide	100 µg/mL, 10 µg/mL, 1 µg/mL	1 µg/mL	Merck Life Science UK Ltd	40, 41
Propranolol	5 µg/mL, 500 ng/mL, 5 ng/mL, 0.5 ng/mL	0.5 ng/mL	VWR International Ltd	42
Metformin	50 µg/mL, 500 ng/mL, 5 ng/mL, 0.5 ng/mL	0.5 ng/mL	VWR International Ltd	43, 44

Supplementary Table 2. Single nucleotide polymorphisms observed in the *ldrA* gene. SNPs identified in the hybrid assemblies of the evolved isolates in comparison to the ancestor. R = test replicate, SNP = single nucleotide polymorphism.

Condition	SNP	Position
Control R3	T ---> C	28
	A ---> G	47
	A ---> G	87
+ Acetaminophen R3	T ---> C	24
	T ---> C	64
	A ---> G	83
+ Titanium dioxide R3	T ---> C	28
	A ---> G	47
	A ---> G	87

Supplementary Table 3. Single nucleotide polymorphisms in the evolved populations. SNPs identified in each sequence (R = test replicate) as predicted by Snippy. The nucleotide(s) in the reference (ref) and comparison (alt) sequences, the locus tag of the gene, and its product are given.

Condition	Ref	Alt	Locus tag	Gene	Product
Ancestor	A	C	00551	*rhsC*	Protein RhsC
Media only R1	A	G	00431		Uncharacterised protein HI_1672
	C	G	00553		Hypothetical protein
	A	G	00553		Hypothetical protein
	T	G			
Media only R2	A	C	00551	*rhsC*	Protein RhsC
Media only R3	A	C	00551	*rhsC*	Protein RhsC
	A	C	03981	*sspA*	Stringent starvation protein A
+ Acetaminophen R1	A	C	00551	*rhsC*	Protein RhsC
	C	G	00553		Hypothetical protein
	A	G	00553		Hypothetical protein
	C	A			
	T	G	03610	*recQ*	ATP-dependent DNA helicase
+ Acetaminophen R2	A	C	00551	*rhsC*	Protein RhsC
	C	G	00553		Hypothetical protein
	A	G	00553		Hypothetical protein
+ Acetaminophen R3	C	G	00553		Hypothetical protein
	A	G	00553		Hypothetical protein
	GCGCC	G	01387	*ygeA*	Putative racemase
+ Ibuprofen R1	A	C	00551	*rhsC*	Protein RhsC
+ Ibuprofen R2	C	G	00553		Hypothetical protein
	A	G	00553		Hypothetical protein
+ Ibuprofen R3	A	C	00551	*rhsC*	Protein RhsC
	A	G	00553		Hypothetical protein
+ Titanium dioxide R1	A	C	00551	*rhsC*	Protein RhsC
	C	G	00553		Hypothetical protein
	A	G	00553		Hypothetical protein
+ Titanium dioxide R2	A	C	00551	*rhsC*	Protein RhsC
	A	G	00553		Hypothetical protein
+ Titanium dioxide R3	A	C	00551	*rhsC*	Protein RhsC
	C	G	00553		Hypothetical protein
	A	G	00553		Hypothetical protein
+ Propranolol R1	A	C	00551	*rhsC*	Protein RhsC
	T	G			
+ Propranolol R2	A	C	00551	*rhsC*	Protein RhsC
+ Propranolol R3	A	C	00551	*rhsC*	Protein RhsC
	A	G	00553		Hypothetical protein
	G	A			
+ Metformin R1	A	C	00551	*rhsC*	Protein RhsC
	C	G	00553		Hypothetical protein
	A	G	00553		Hypothetical protein
+ Metformin R2	A	C	00551	*rhsC*	Protein RhsC
	A	G	00553		Hypothetical protein
	G	T			
+ Metformin R3	None				
