# Supplementary material for: Non-antibiotic pharmaceuticals are toxic against Escherichia coli with no evolution of cross-resistance to antibiotics
Source: NPJ Antimicrob Resist. 2024 Apr 15;2:11. doi: 10.1038/s44259-024-00028-5 (PMC11721113; doi:10.1038/s44259-024-00028-5)
Supplement: Supplementary file 2 — Reporting Summary [file 44259_2024_28_MOESM2_ESM.pdf]

nature portfolio

Corresponding author(s): Alan McNally

Last updated by author(s): 21/1/2024

Reporting Summary

Nature Portfolio wishes to improve the reproducibility of the work that we publish. This form provides structure for consistency and transparency in reporting. For further information on Nature Portfolio policies, see our Editorial Policies and the Editorial Policy Checklist.

Statistics

For all statistical analyses, confirm that the following items are present in the figure legend, table legend, main text, or Methods section.

n/a	Confirmed	
☐	☒	The exact sample size (*n*) for each experimental group/condition, given as a discrete number and unit of measurement
☐	☒	A statement on whether measurements were taken from distinct samples or whether the same sample was measured repeatedly
☐	☒	The statistical test(s) used AND whether they are one- or two-sided<br>*Only common tests should be described solely by name; describe more complex techniques in the Methods section.*
☐	☒	A description of all covariates tested
☐	☒	A description of any assumptions or corrections, such as tests of normality and adjustment for multiple comparisons
☐	☒	A full description of the statistical parameters including central tendency (e.g. means) or other basic estimates (e.g. regression coefficient) AND variation (e.g. standard deviation) or associated estimates of uncertainty (e.g. confidence intervals)
☐	☒	For null hypothesis testing, the test statistic (e.g. *F*, *t*, *r*) with confidence intervals, effect sizes, degrees of freedom and *P* value noted<br>*Give P values as exact values whenever suitable.*
☒	☐	For Bayesian analysis, information on the choice of priors and Markov chain Monte Carlo settings
☒	☐	For hierarchical and complex designs, identification of the appropriate level for tests and full reporting of outcomes
☐	☒	Estimates of effect sizes (e.g. Cohen's *d*, Pearson's *r*), indicating how they were calculated

Our web collection on statistics for biologists contains articles on many of the points above.

Software and code

Policy information about availability of computer code

Data collection	No software was used
Data analysis	Long-read bse calling was conducted using Guppy (v6.0.1). Reads were filtered using Filtlong (v0.2.1) using a cut-off of 600000000 target bases and demultiplexed using qcat (v1.1.0). Hybrid assemblies were then generated using Unicycler (v0.4.8-beta) in bold mode. Panaroo (v1.2.10) was used to generate gene presence/absence and core gene alignment files with the latter used to construct a maximum likelihood tree with IQ-TREE (v2.2.0.3). The tree and gene presence/absence data were visualised in Phandango [47] to look for differential gene presence patterns across the evolved isolates. A custom ABRicate (v0.8) database was used to investigate the presence and identity of the ldrA gene across the evolved isolates. The presence of SNPs was analysed using snippy (v4.3.6). Potential movement of insertion sequence (IS) elements was investigated using ISEScan (v1.7.2.3), ISFinder [48], and a custom ABRicate (v0.8) database, with sequences interrogated in Unipro UGENE (v47.0).<br>Differential gene expression was quantified using Kallisto (v0.48.0) A long-read assembly of the ancestral E. coli, annotated using Prokka (v1.14.6), was used as a reference. The annotated assembly was processed using genbank_to_kallisto.py (https://github.com/AnnaSyme/genbank_to_kallisto.py). GNU parallel [49] was used for job parallelization. Differential gene expression was analyzed in Degust (v4.1.1) with a false discovery rate threshold of $p < 0.05$ and an absolute log fold change of at least 1.<br>Area under the curve measurements were calculated using numpy.trapz in Python (v3.9.10).

For manuscripts utilizing custom algorithms or software that are central to the research but not yet described in published literature, software must be made available to editors and reviewers. We strongly encourage code deposition in a community repository (e.g. GitHub). See the Nature Portfolio guidelines for submitting code & software for further information.

Data

Policy information about availability of data

All manuscripts must include a data availability statement. This statement should provide the following information, where applicable:

- Accession codes, unique identifiers, or web links for publicly available datasets
- A description of any restrictions on data availability
- For clinical datasets or third party data, please ensure that the statement adheres to our policy

The datasets generated and analysed during the current study are available from NCBI BioProject with accession PRJNA1005239.

Research involving human participants, their data, or biological material

Policy information about studies with human participants or human data. See also policy information about sex, gender (identity/presentation), and sexual orientation and race, ethnicity and racism.

Reporting on sex and gender	Not relevant and no collected
Reporting on race, ethnicity, or other socially relevant groupings	Not relevant and not collected
Population characteristics	Not relevant and not collected
Recruitment	Not relevant and not collected
Ethics oversight	Not relevant

Note that full information on the approval of the study protocol must also be provided in the manuscript.

Field-specific reporting

Please select the one below that is the best fit for your research. If you are not sure, read the appropriate sections before making your selection.

☒ Life sciences ☐ Behavioural & social sciences ☐ Ecological, evolutionary & environmental sciences

For a reference copy of the document with all sections, see nature.com/documents/nr-reporting-summary-flat.pdf

Life sciences study design

All studies must disclose on these points even when the disclosure is negative.

Sample size	Sample sizes are not relevant to this study
Data exclusions	No data was excluded
Replication	All data in the manuscript was repeated in triplicate including the transcriptomic data
Randomization	Randomization is not relevant to our study which was comparing effects on bacteria with and without a treatment
Blinding	Blinding is not relevant to our study which was comparing effects on bacteria with and without a treatment

Reporting for specific materials, systems and methods

We require information from authors about some types of materials, experimental systems and methods used in many studies. Here, indicate whether each material, system or method listed is relevant to your study. If you are not sure if a list item applies to your research, read the appropriate section before selecting a response.

Materials & experimental systems

n/a	Involved in the study
☒	☐ Antibodies
☒	☐ Eukaryotic cell lines
☒	☐ Palaeontology and archaeology
☒	☐ Animals and other organisms
☒	☐ Clinical data
☒	☐ Dual use research of concern
☒	☐ Plants

Methods

n/a	Involved in the study
☒	☐ ChIP-seq
☒	☐ Flow cytometry
☒	☐ MRI-based neuroimaging

Plants

Seed stocks	Not relevant
Novel plant genotypes	Not Relevant
Authentication	Not relevant
